# Protective role of melatonin against adipose-hepatic metabolic comorbidities in experimentally induced obese rat model

**DOI:** 10.1371/journal.pone.0260546

**Published:** 2021-12-08

**Authors:** Mary J. Obayemi, Christopher O. Akintayo, Adesola A. Oniyide, Ayodeji Aturamu, Olabimpe C. Badejogbin, Chukwubueze L. Atuma, Azeezat O. Saidi, Hadiza Mahmud, Kehinde S. Olaniyi

**Affiliations:** 1 Department of Physiology, College of Medicine and Health Sciences, Afe Babalola University, Ado-Ekiti, Nigeria; 2 Department of Physiology, Benjamin Carson School of Medicine, Babcock University, Ilishan-Remo, Nigeria; Max Delbruck Centrum fur Molekulare Medizin Berlin Buch, GERMANY

## Abstract

**Background:**

Adipose and hepatic metabolic dysfunctions are critical comorbidities that also aggravate insulin resistance in obese individuals. Melatonin is a low-cost agent and previous studies suggest that its use may promote metabolic health. However, its effects on some comorbidities associated with obesity are unknown. Herein, we investigated the hypothesis that melatonin supplementation would attenuate adipose-hepatic metabolic dysfunction in high fat diet (HFD)-induced obesity in male Wistar rats.

**Materials and methods:**

Twenty-four adult male Wistar rats (n = 6/group) were used: Control group received vehicle (normal saline), obese group received 40% high fat diet, melatonin-treated group received 4 mg/kg of melatonin, and obese plus melatonin group received 40% HFD and melatonin. The treatment lasted for 12 weeks.

**Results:**

HFD caused increased food intake, body weight, insulin level, insulin resistance and plasma and liver lipid but decreased adipose lipid. In addition, HFD also increased plasma, adipose and liver malondialdehyde, IL-6, uric acid and decreased Glucose-6-phosphate dehydrogenase, glutathione, nitric oxide and circulating obestatin concentration. However, these deleterious effects except food intake were attenuated when supplemented with melatonin.

**Conclusion:**

Taken together, the present results indicate that HFD exposure causes adipose-hepatic metabolic disturbance in obese animals, which are accompanied by oxidative stress and inflammation. In addition, the present results suggest that melatonin supplementation attenuates adipose-hepatic metabolic dysfunction, accompanying obesity by suppression of oxidative stress/inflammation-dependent mechanism and increasing circulating obestatin.

## 1. Introduction

Obesity has become a global epidemic in the twenty-first century. Overweight individuals aged 18 and above accounted for more than 1.9 billion people in 2016. Over 650 million adults in this group were overweight or obese, 39% were overweight while over 13% of the group were obese. Thus, obesity prevalence nearly tripled globally between 1975 and 2016. In 2020, 39 million children under the age of five were overweight or obese [1]. Obesity is a multifaceted, diverse disease influenced by hormones, nutritional consumption, sedentary lifestyles, physical activity, genetics, and environmental variables [2, 3]. This metabolic disease is rising with comorbidities, including non-alcoholic fatty liver disease (NAFLD) that reduce life quality and expectancy, primarily due to cardiometabolic problems [4]. The pathogenesis of cardiometabolic dysfunctions are low-grade systemic inflammation and insulin resistance caused by cytokines. These cytokines are released by excess adipose tissue in the body, especially in the visceral site [5–7].

In the quest for effective control of obesity, four hormones were discovered to have a link; insulin, leptin, ghrelin and obestatin and the growth hormone secretagogue receptor (GHS-R) [8]. Insulin is secreted in the pancreas by β-cells islets of Langerhans and has a variety of biological functions, such as body weight regulation and glucose homeostasis [9, 10]. However, insulin resistance arises as a result of obesity and obesity-related dysfunctions including type 2 diabetes mellitus (T2DM) and cardiovascular disorders. Hyperinsulinemia, resulting from either hypersecretion or reduced insulin clearance, is a symptom of obesity and can lead to IR sensitivity [11, 12]. Leptin was the initial cytokine derived from adipose tissue linked with energy balance [13]) and is an anorexigenic hormone produced mostly by adipose tissue. Leptin synthesis and secretion into circulation are increased when fat depots expand in conjunction with a favorable energy balance [14]. It has been observed that obesity promotes hyperleptinemia and leptin resistance [15]. Ghrelin initially identified as an endogenous ligand of the growth hormone secretagogue receptor (GHSR1) is an orexigenic peptide. It is derived primarily from the stomach and a peripheral signal that promotes food intake [16]. Obese people have lower plasma ghrelin level and their meal-related ghrelin variations are similarly affected [17]. A study showed that there was decreased ghrelin sensitivity after the administration of leptin, implying that the increased leptinemia observed in obesity is responsible for the resistance of ghrelin [18].

Associated with the pathophysiology of obesity-related metabolic dysfunctions are hyperleptinemia and hyperinsulinemia, and body adiposity in obesity is relative to insulin and leptin levels in the circulation. Ghrelin dysregulation can also occur in obesity and play a role in mediating some of the pathological signs and symptoms [19]. Obestatin is a 23-amino acid anorexic hormone, a peptide that is involved in appetite control and long-term energy regulation together with ghrelin [20]. Ghrelin and obestatin are both derived from a single preproghrelin gene and produced by post-translation modification of preproghrelin but obestatin has a distinct terminus [21]. Hence, it is reported to have opposite effect on food intake as ghrelin [22]. It is an anorexigenic hormone that suppresses appetite and gastrointestinal motility and modulates growth hormone and lipid metabolism [23, 24]. However, previous studies have reported that obestatin acts as antagonist to the actions of ghrelin on appetite, food intake, gastric emptying and the secretion of growth hormone [25, 26]. Zhao *et al*., also reported that obestatin is reduced in obese humans [27].

Melatonin is a hormone secreted by the pineal gland in the dark hours via the control of the suprachiasmatic nucleus of the hypothalamus. It is associated with many physiological roles in the central nervous system, sleep and wakefulness cycles, energy metabolism and thermoregulation, immune and endocrine regulation among others [28]. Melatonin is the significant mediator molecule in the incorporation of the cyclic environment and the circadian distribution of physiological and cognitive processes, as well as the optimization of energy hemostasis and regulation of body weight, which are important for a healthy metabolism [29]. The islets of Langerhans of the pancreas are important sites of the action of melatonin where it stimulates the synthesis and secretion of insulin and glucagon synthesis in reference to the regulation of energy metabolism. The melatonin receptors MT1 and/or MT2- facilitated the action of melatonin decreasing the glucose-stimulated insulin secretion (GSIS) in the isolated pancreatic islets and insulinoma beta cells in rats [30, 31]. Through the regulation of GLUT4 expression or triggering the insulin signaling pathway, melatonin functions in potentiating central and peripheral action of insulin. Thus, it induces, via its G-protein-coupled membrane receptors, the phosphorylation of the insulin receptor and its intracellular substrates. It has also been considered that melatonin’s association with all the physiological processes typical of the daily activity-wakefulness/rest-sleep rhythm may impact body weight and possibly contribute to energy homeostasis [28, 32]. However, information on the role of melatonin in obesity-associated adipose-hepatic metabolic dysregulation is lacking. The present study was therefore designed to investigate the role of melatonin on adipose-hepatic metabolic perturbations in obese male Wistar rats. The study in addition determined the probable involvement of obestatin.

## 2. Materials and methods

### 2.1. Animals

All experimental protocols for this study were conducted in accordance with the National Institutes of Health Guide for the Care and Use of Laboratory Animals and was approved by the Institutional Ethical Review Board of Afe Babalola University, Nigeria (ABUADERC/10/2020), and every effort was made to minimize both the number of animals used and their suffering. Twenty-four male Wistar rats weighing 170–200 g were procured from the animal house of the College of Health Sciences, Afe Babalola University, Nigeria. Rats had unrestricted access to standard rat chow and tap water. After 2 weeks of acclimatization, the animals were randomly assigned into four groups (n = 6 per group). Rats were maintained in a colony under standard environmental conditions of temperature (22–26°C), relative humidity (50–60%), and 12-hour dark/light cycle.

### 2.2. Treatment

Control (CTL) received diet and distilled water (vehicle; *po*), Melatonin-treated group (MLT-treated) received melatonin (4 mg/kg body weight; Sigma-Aldrich, St Louis, MI), Obese group (OBS) received 40% high fat diet (HFD) and Obese with melatonin-treated group (OBS+MLT-treated) received combination of high fat and melatonin daily. Animals were treated with melatonin between 8:00–10:00 am and obesity was induced by exposing the animals to 40% HFD ad libitum as previously described [33] The administration lasted for 12 weeks. Initial and final body weights were determined, and body weight gain was estimated. In addition, daily food and water consumptions were monitored for week 0 (initial) and week 12 (final) by subtracting the left-over food and water after 24 h from the food and water that were introduced to the animals. The changes in food and water consumptions were estimated by subtracting the initial consumption from the final consumption.

### 2.3. Sample preparation

After 12 weeks of administration, the animals were fasted overnight for 12 h. Thereafter, the animals were anesthetized by intraperitoneal injection of 50 mg/kg *b*.*w*. of sodium pentobarbital. Cardiac puncture was used for the collection of blood into the heparinized tube and blood was centrifuged at room temperature for 5 mins at 3000 rpm. Plasma was decanted and stored frozen until when it was needed for the biochemical analysis.

### 2.4. Preparation of liver and adipose tissue homogenates

After weighing the liver and visceral fat, 100 mg section of each tissue was carefully removed and homogenized with a glass homogenizer in phosphate buffer solution, centrifuged at 10000 rpm for 10 min at 4°C.

### 2.5. Blood glucose and insulin resistance (IR)

Fasting blood glucose was determined with a hand-held glucometer (ONETOUCH®-LifeScan, Inc., Milpitas, CA, USA). Insulin resistance was estimated using the Homeostatic model assessment for IR (HOMA-IR = fasting glucose (mmol/l) _*_ fasting insulin (μU/l)/22.5) [34, 35].

### 2.6. Biochemical assays

#### 2.6.1. Plasma insulin

The plasma level of insulin was determined with Rat ELISA kits obtained from Calbiotech Inc. (Cordell Ct., El Cajon, CA 92020, USA) in compliance with the manufacturer’s procedures and based on the direct sandwich technique in which two monoclonal antibodies are directed against separate antigenic determinants on the insulin molecule.

#### 2.6.2. Obestatin

Obestatin concentration was determined in the plasma using Rat ELISA kits obtained from Calbiotech Inc. (El Cajon, USA) in compliance with the manufacturer’s assay procedure.

#### 2.6.3. Lipid profile

Concentration of triglycerides (TG) and total cholesterol (TC) were estimated in the plasma, liver and adipose tissue homogenates by standardized colorimetric methods using reagents obtained from Fortress Diagnostics Ltd. (Antrim, UK).

#### 2.6.4. Oxidative stress markers

Malondialdehyde (MDA) was determined from the plasma, liver and adipose tissue homogenate by standard non-enzymatic spectrophotometric method using assay kits from Randox Laboratory Ltd. (Co. Antrim, UK). This method was carried out as previously described [35], whereas Glutathione (GSH) was determined using non-enzymatic spectrophotometric method with assay kits obtained from Oxford Biomedical Research Inc. (Oxford, USA). Glutathione was determined by spectrophotometric method based on the oxidation of GSH in the sample by the sulfhydryl reagent 5,5′-dithio-bis (2-nitrobenzoic acid) (DTNB) to form the yellow derivative 5′-thio-2-nitrobenzoic acid (TNB), measured at 412 nm. While Glucose-6-phosphate dehydrogenase (G6PD) activity was determined from the plasma, liver and adipose tissue using standard spectrophotometric method with assay kits obtained from Calbiotech Inc. (El Cajon, USA).

#### 2.6.5. Interleukin-6 (IL-6), nitric oxide and uric acid concentration

Plasma, liver and adipose tissue concentration of IL-6 was determined by the quantitative standard sandwich ELISA technique using monoclonal antibody specific for these parameters with rat kits obtained from Elabscience Biotechnology Inc. (Wuhan, Hubei, P.R.C., China). Nitric oxide was assayed spectrophotometrically by measuring the accumulation of its stable degradation products, nitrate and nitrite using kits from Oxford Biomedical Research Inc., (Oxford, UK). This kit employs the NADH-dependent enzyme nitrate reductase for conversion of nitrate to nitrite prior to the quantification of nitrite using Griess reagent—thus providing for accurate determination of total NO production. Furthermore, uric acid uric concentration was estimated by non-enzymatic colorimetric method using assay kits from Randox Laboratory Ltd. (Co. Antrim, UK) and in compliance with the manufacturer’s assay procedures.

### 2.7. Statistical analysis

Shapiro-Wilk test was used to confirm the data distribution, and the data were normally distributed. All data were expressed as means ± SD. Statistical group analysis was performed using the Graphpad prism 5. One-way ANOVA was used to compare the mean values of variables among the groups. Bonferroni’s test was used for *post hoc* analysis. Statistically significant differences were accepted at p less than 0.05.

## 3. Results

### 3.1. Effects of melatonin on food intake, water intake and body weight in HFD-induced obese rats

There was a significant increase (p<0.05) in food intake in obese and OBS+MLT-treated rats compared to the control group. Supplementation with melatonin did not significantly decrease the food intake as shown in OBS+MLT-treated rats compared with obese rats. In addition, body weight was increased in obese rats when compared to the control group. However, melatonin decreased the body weight. There was no alteration in water intake in all the experimental groups compared to the control group (Table 1).

**Table 1:** Melatonin attenuates excess body weight but not food intake in HFD-induced obese animals.

GROUPS	CTL	MLT	OBS	OBS+MLT
**Food intake (g/day)**				
**Initial**	25.22 ± 0.81	33.15 ± 2.30	30.59 ± 4.24	31.85 ± 2.27
**Change**	8.01 ± 2.71	5.33 ± 1.79	19.21 ± 3.77*	14.42 ± 0.35*
**Water intake (mL/day)**				
**Initial**	32.62 ± 1.47	27.79 ± 3.16	26.63 ± 3.43	35.63 ± 3.43
**Change**	7.34 ± 2.52	5.44 ± 10.19	6.18 ± 5.44	5.86 ± 3.88
**Body weight (g)**				
**Initial**	172.71 ± 6.41	174.93 ± 8.12	171.00 ± 6.65	171.43 ± 5.70
**Gain**	44.40 ± 6.70	36.67 ± 9.30	75.87 ± 4.72*	26.69 ± 3.62^#^

Data are expressed as mean ± SD. n = 6 and analyzed by one-way ANOVA followed by Bonferroni *post hoc test*. (*p<0.05 vs. CTL; #*p*<0.05 vs. OBS). Control (CTL); Melatonin (MLT); Obesity (OBS).

### 3.2. Effects of melatonin on glucose homeostasis in HFD-induced obese rats

**Fig 1:**
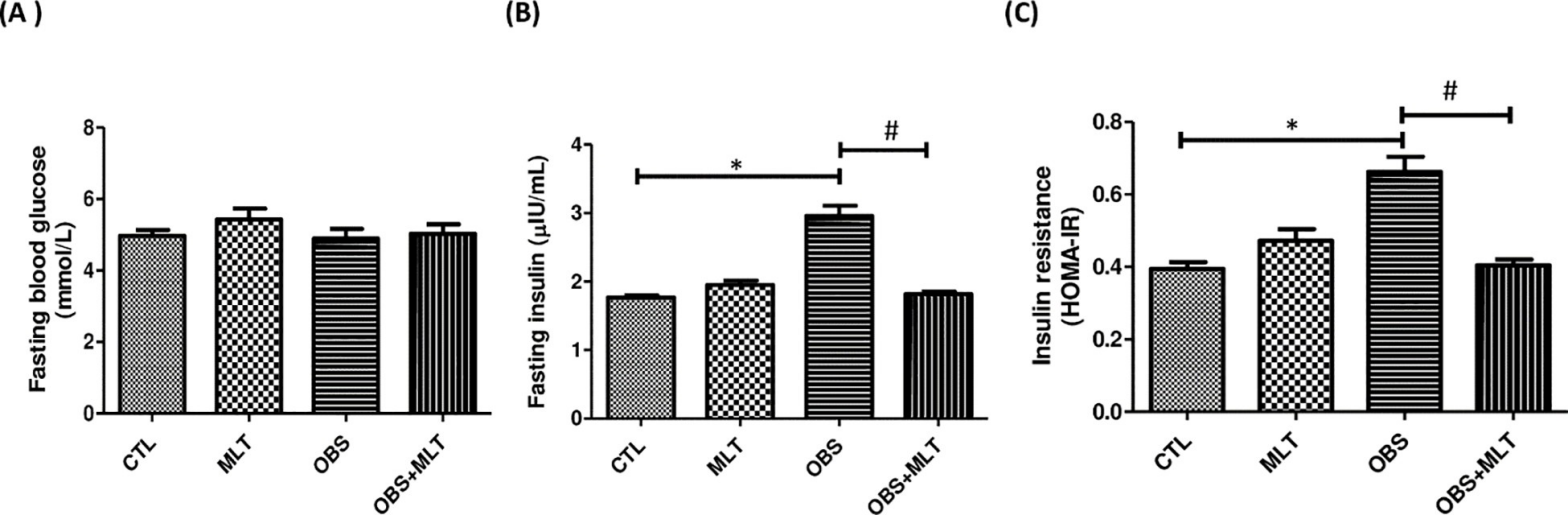
Effects of melatonin on blood glucose (A), insulin (B) and insulin resistance (C) HFD-induced obese animals. Data are expressed as mean ± SD. n = 6 and analyzed by one-way ANOVA followed by Bonferroni *post hoc test*. (*p<0.05 vs. CTL; #*p*<0.05 vs. OBS). Control (CTL); Melatonin (MLT); Obesity (OBS).

There was a significant increase (*p*<0.05) in fasting plasma insulin but no alteration in blood glucose in obese group compared to the control group. However, supplementation with melatonin decreased the fasting plasma insulin in OBS+MLT group compared to the untreated obese group. Similarly, insulin resistance was observed in the obese animals compared with control animals. Administration of melatonin significantly reduced insulin resistance in OBS+MLT group compared to the untreated obese group (Fig 1).

### 3.3. Effects of melatonin on plasma, adipose and liver triglyceride and total cholesterol in HFD-induced obese rats

**Fig 2:**
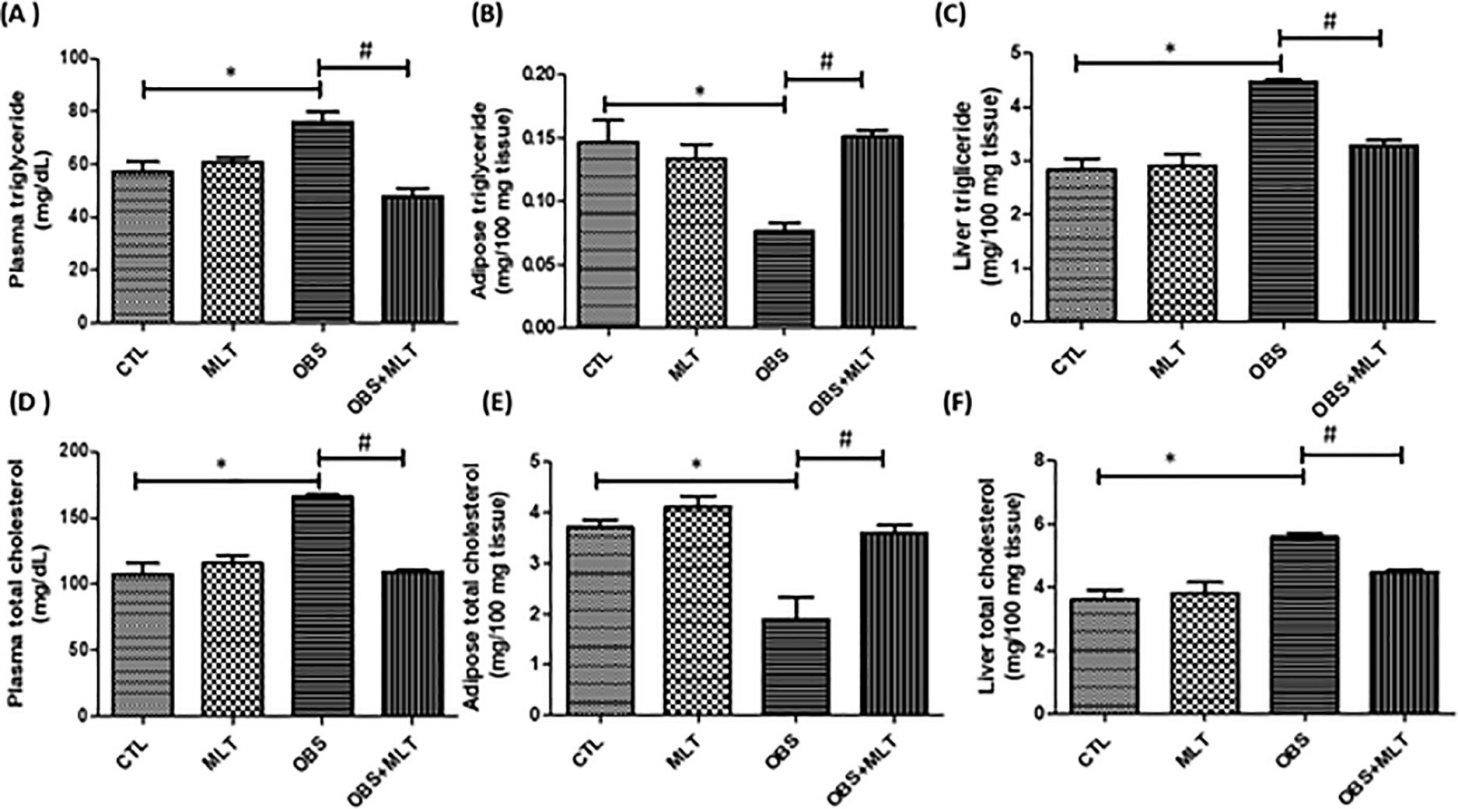
Effects of melatonin on plasma, adipose and liver triglyceride (A-C) and total cholesterol (D-F) in HFD-induced obese rats. Data are expressed as mean ± SD. n = 6 and analyzed by one-way ANOVA followed by Bonferroni *post hoc test*. (*p<0.05 vs. CTL; #*p*<0.05 vs. OBS). Control (CTL); Melatonin (MEL); Obesity (OBS); Total cholesterol (TC).

There was a significant increase (*p*<0.05) in plasma and liver TG and TC but a decrease in adipose triglyceride and total cholesterol in obese group compared to the control group. However, supplementation with melatonin decreased the plasma and liver TG and TC and as well increased the TG and TC concentrations in the adipose tissue of OBS+MLT group compared to the untreated obese group (Fig 2).

### 3.4. Effect of melatonin on malondialdehyde in HFD-induced obese rats

**Fig 3:**
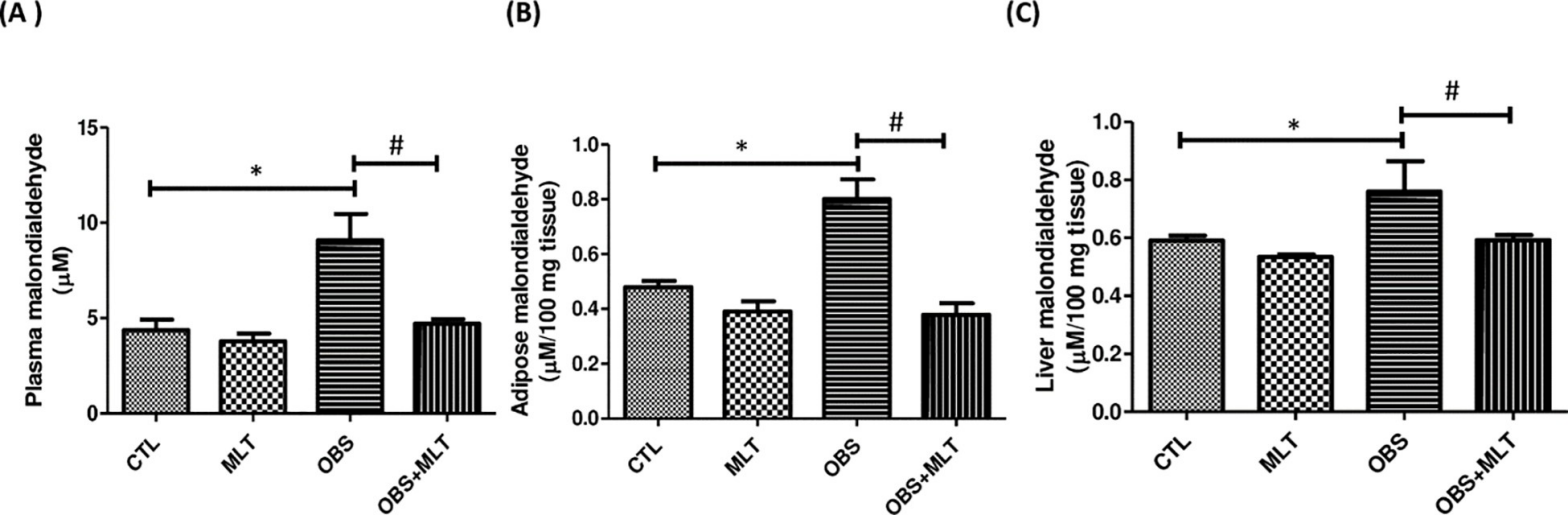
Effect of melatonin on plasma, adipose and liver malondialdehyde (A-C) in HFD-induced obese rats. Data are expressed as mean ± SD. n = 6 and analyzed by one-way ANOVA followed by Bonferroni *post hoc test*. (*p<0.05 vs. CTL; #*p*<0.05 vs. OBS). Control (CTL); Melatonin (MLT); Obesity (OBS).

There was a significant increase (*p*<0.05) in plasma, adipose and liver MDA in obese group compared to the control group. However, supplementation with melatonin decreased the plasma, adipose and liver MDA in OBS+MLT group compared to the untreated obese group (Fig 3).

### 3.5. Effect of melatonin on G6PD and GSH in HFD-induced obese rats

**Fig 4:**
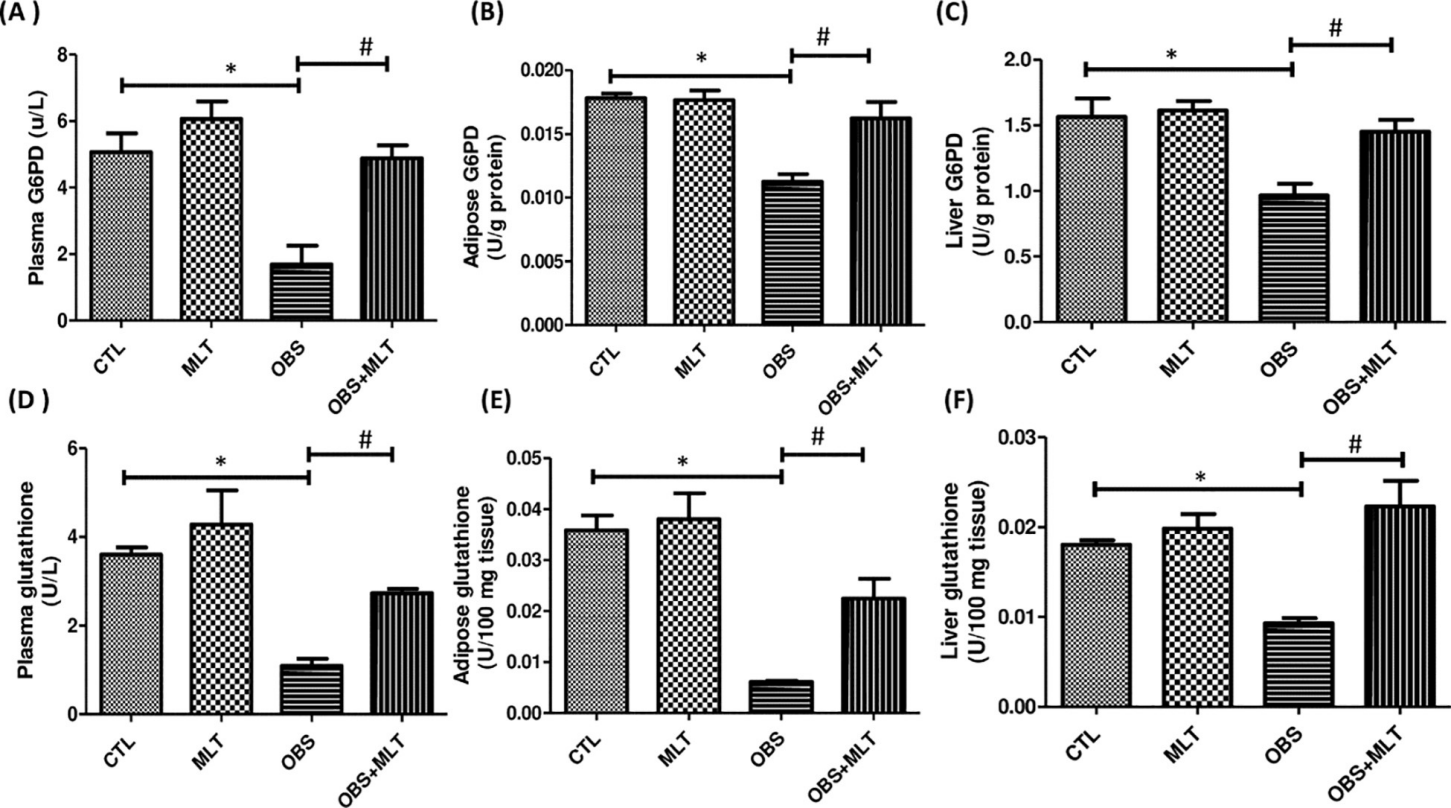
Effects of melatonin on plasma, adipose and liver Glucose-6-phosphate dehydrogenase (A-C) and glutathione (D-F) in HFD-induced obese rats. Data are expressed as mean ± SD. n = 6 and analyzed by one-way ANOVA followed by Bonferroni *post hoc test*. (*p<0.05 VS. CTL; ^#^*p*<0.05 VS. OBS). Control (CTL); Melatonin (MLT); Obesity (OBS); Glucose 6 phosphate dehydrogenase (G6PD); Glutathione (GSH).

There was a significant decrease (*p*<0.05) in plasma, adipose and liver G6PD activity and glutathione concentration in obese group compared to the control group. Nonetheless, supplementation with melatonin increase the plasma, adipose and liver G6PD and glutathione concentration in OBS+MLT group compared to the untreated obese group (Fig 4).

### 3.6. Effects of melatonin on IL-6 and uric acid concentration in HFD-induced obese rats

**Fig 5:**
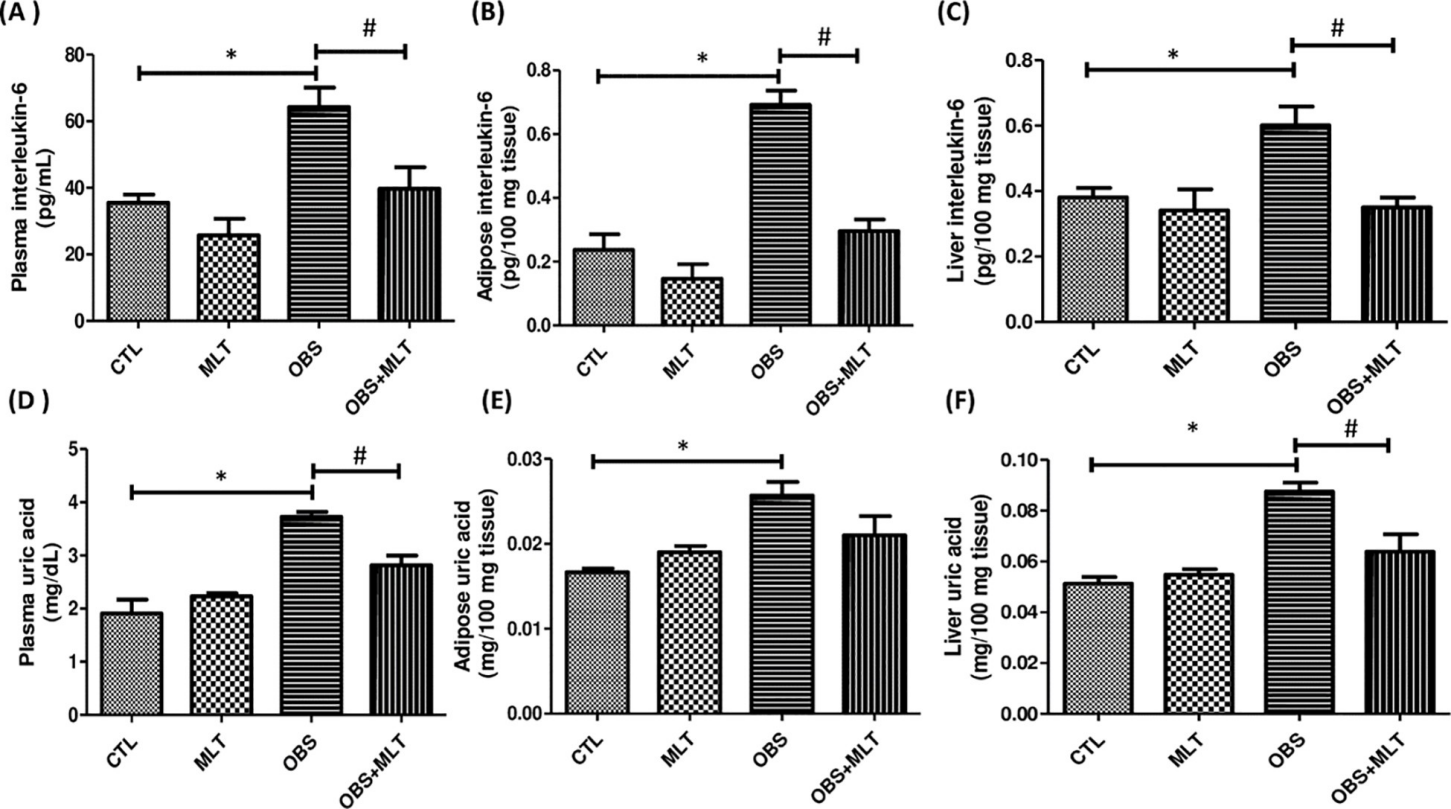
Effects of melatonin on plasma, adipose and liver interleukin-6 (A-C) and uric acid concentration (D-F) HFD-induced obese rats. Data are expressed as mean ± SD. n = 6 and analyzed by one-way ANOVA followed by Bonferroni *post hoc test*. (*p<0.05 VS. CTL; ^#^*p*<0.05 VS. OBS). Control (CTL), Melatonin (MLT), Obesity (OBS).

There was a significant increase (*p*<0.05) in plasma, adipose and liver IL-6 and uric acid concentration in obese group compared to the control group. However, supplementation with melatonin decrease the plasma and liver but not adipose uric acid concentration in OBS+MLT group compared to the untreated obese group (Fig 5)

### 3.7. Effects of melatonin on nitric oxide concentration in HFD-induced obese rats

**Fig 6:**
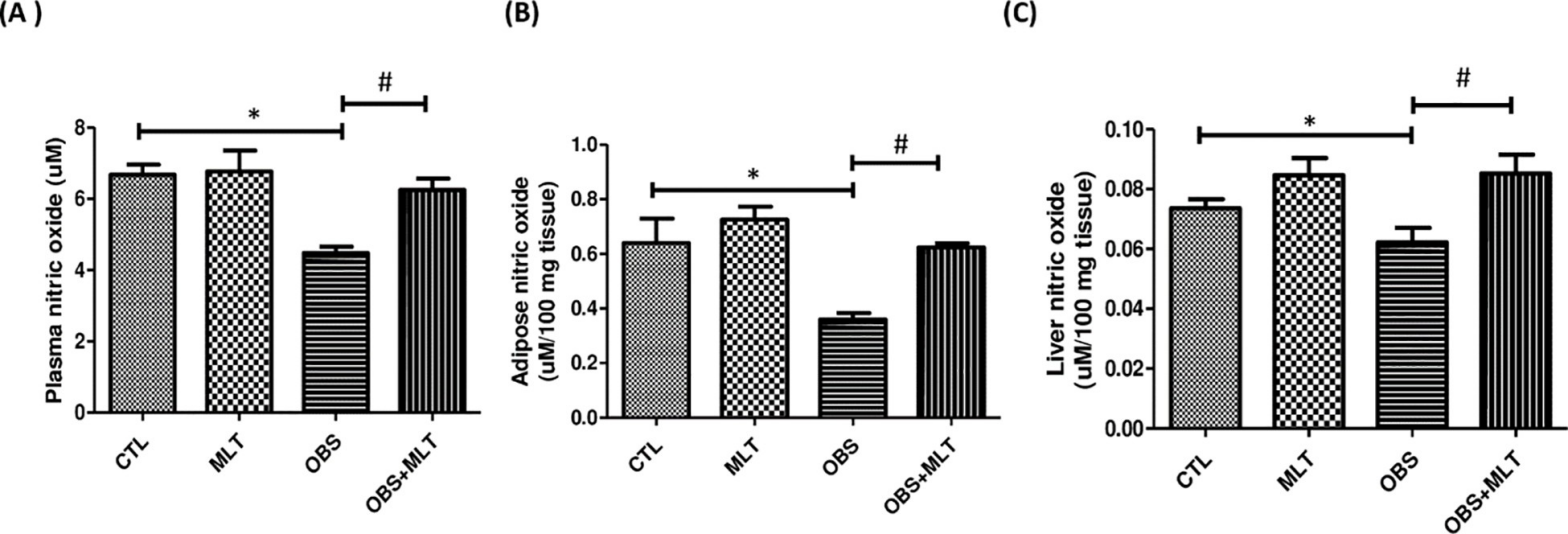
Effects of melatonin on plasma, adipose and liver nitric oxide concentration (A-C) in HFD-induced obese rats. Data are expressed as mean ± SD. n = 6 and analyzed by one-way ANOVA followed by Bonferroni *post hoc test*. (*p<0.05 VS. CTL; ^#^*p*<0.05 VS. OBS). Control (CTL); Melatonin (MLT); Obesity (OBS).

There was a significant reduction (*p*<0.05) in plasma, adipose and liver nitric oxide concentration in obese group compared to the control group. However, supplementation with melatonin increased the plasma, adipose and liver nitric oxide concentration in OBS+MLT group compared to the untreated obese group (Fig 6).

### 3.8. Effects of melatonin on obestatin level in HFD-induced obese rats

**Fig 7:**
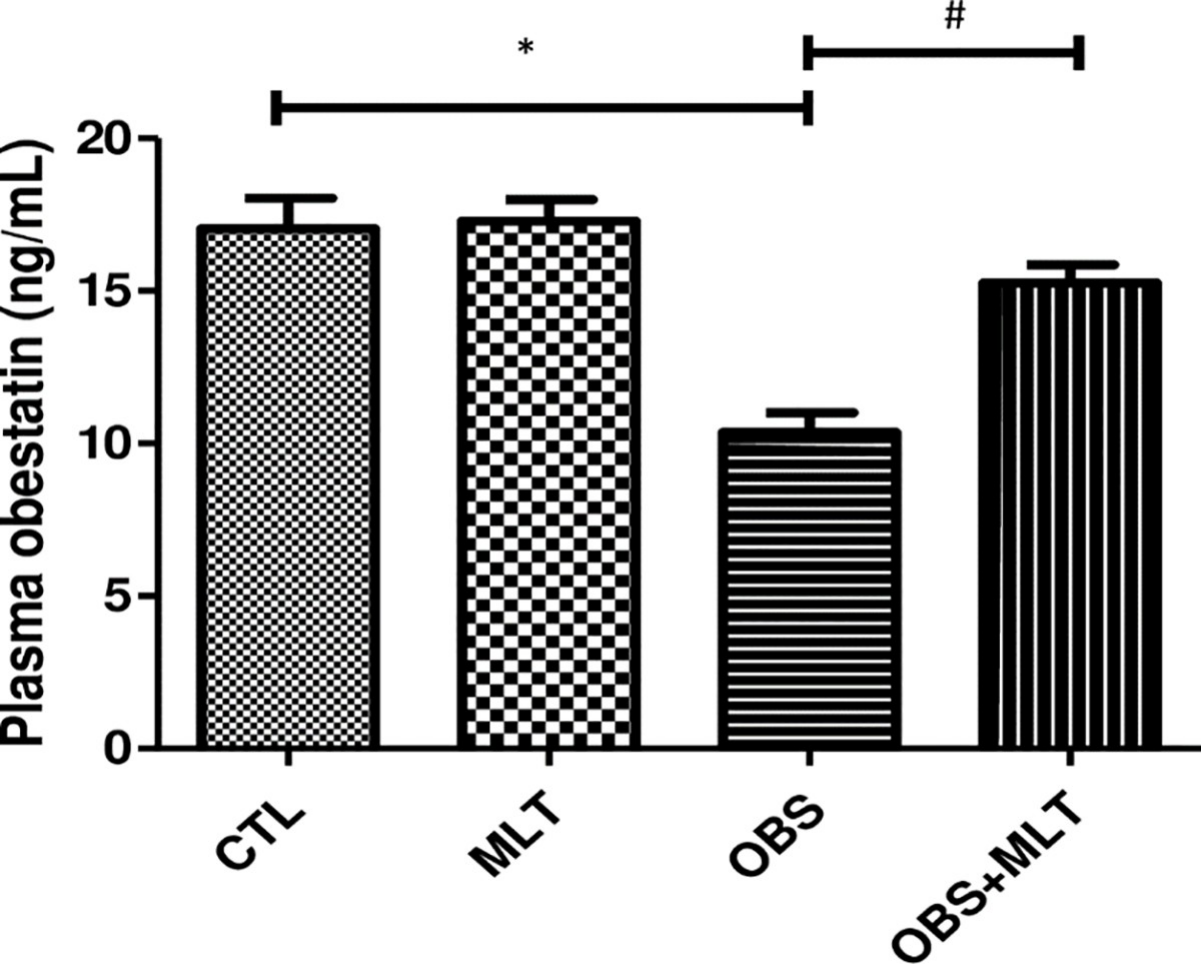
Effects of melatonin on circulating obestatin concentration in HFD-induced obese rats. Data are expressed as mean ± SD. n = 6 and analyzed by one-way ANOVA followed by Bonferroni *post hoc test*. (*p<0.05 VS. CTL; ^#^*p*<0.05 VS. OBS). Control (CTL), Melatonin (MLT), Obesity (OBS).

There was a significant decrease (p<0.05) in the level of plasma obestatin concentration in obese animal when compared to the control animal. However, supplementation with melatonin significantly increased the obestatin level in animal with obesity (Fig 7).

## 4. Discussion

The data from the present study demonstrated that melatonin reversed the adipose-hepatic metabolic comorbidities associated with obesity in male Wistar rats by suppression of oxidative stress, inflammation and increasing circulating obestatin. Earlier studies have demonstrated a significant decrease in the level of obestatin in obese children [36], and the present observation that revealed a significant decrease in the circulating levels of obestatin in obese animals compared to control group is consistent with previous studies. In addition, Ren *et al*., reported that the levels of obestatin were significantly lower in obese subjects and correlated negatively with body mass index (BMI), insulin, glucose and insulin resistance [37]. However, as revealed in the results of the present studies, in addition to decreased obestatin level, obesity is also characterized with insulin resistance, hyperinsulinemia and excess body weight, which are consistent with previous observations [23, 37]. Besides, the present results also showed an increase in food intake in obese animals compared to the control group, which might contribute to excess body weight possibly due to reduced energy utilization resulting from insulin resistance. As already demonstrated, HFD causes insulin resistance in experimental rodents [38, 39].

Furthermore, compensatory hyperinsulinemia observed in obese rats might contribute to normal blood glucose. However, evidence exists that hyperinsulinemia signals oxidative stress, especially in combination with insulin resistance causing adipose tissue inflammation that characterized obesity [40–42]. In addition, obesity is considered a syndrome of excessive visceral adiposity and is linked with metabolic dysfunctions. Metabolic dysfunctions are characterized by cardiovascular and diabetes risk factors such as abdominal adiposity, hypertension, reduction in high‐density lipoprotein (HDL), increased triglycerides and glucose intolerance [43]. In this study, there was a significant increase in plasma and liver TG and TC with corresponding decrease in adipose TG and TC in obese group compared to the control group, which might lead to hepatic lipotoxicity that triggered oxidative stress in obese animals as shown by elevated hepatic lipid peroxidation (MDA) with a decrease in G6PD/GSH-dependent antioxidant capacity. Previous studies have documented that a decrease in obestatin could also contribute to increase in TC with consequent oxidative stress [44–46]. Therefore, in this study obesity-induced hepatic oxidative stress is associated with a decrease in circulating level of obestatin and excessive lipolysis that led to reduction in adipose TG and TC.

In addition, the present study showed a significant increase in plasma, adipose and liver IL-6 and uric acid concentration and a significant reduction in plasma and adipose nitric oxide concentration in obese group compared to the control group. These observations are consistent with earlier studies, including a recent study from our laboratory animals which demonstrates that metabolic related syndrome such as obesity causes inflammation in the metabolic tissues, particularly the adipose and hepatic tissues [47, 48] and these are well documented pathological features of non-alcoholic fatty liver disease [47, 49]. This therefore suggests obesity as a predictor of fatty liver disease, which may become one of the common reasons for liver transplantations by 2030 especially in developed countries [49, 50]. Other studies have also reported that obestatin could be protective against oxidative stress and inflammation [51, 52]. Therefore, decrease level of obestatin might in part contribute to adipose/hepatic inflammation that characterized obese animals compared to the control group.

Interestingly, this study also showed that melatonin supplementation reduced the body weight of obese rats while elevating their levels of obestatin, though without a significant decrease in food intake compared to the untreated obese group. The treatment with melatonin also decreased the plasma and liver triglyceride and total cholesterol in OBS+MLT group compared to the untreated obese group. In addition, the elevated fasting plasma insulin and insulin resistance were reversed by melatonin supplementation, which might be due to increase in insulin sensitivity as earlier reported by McHugh and Cheng that administration of melatonin improves insulin sensitivity and insulin level [53]. This possibly improved glucose/lipid metabolism and thus prevents excess energy storage/visceral adiposity that constitutes excess body weight gain. This observation seems similar to a number of studies that demonstrated improved body composition following administration of melatonin [32, 39, 54]. Besides, melatonin has also been reported to modulate cyclic adenosine monophosphate (cAMP) and cyclic gaunosine monophosphate (cGMP levels), which regulate glucose and energy homeostasis [55] corroborating that melatonin promotes body maintenance. Other studies have also shown that administration of melatonin prevented high glucose or lipid levels in pinealectomized rats [56]. However, melatonin in addition to improving body weight, insulin sensitivity also demonstrated antioxidant effect against hepatic and adipose oxidative stress with corresponding decrease in lipid peroxidation and enhancement of G6PD/GSH-dependent antioxidant barrier in obese animals compared to the untreated obese group. Likewise, the administration of melatonin increased the plasma, liver and adipose nitric oxide concentration and decreased the plasma and liver uric acid and IL-6 concentration with corresponding decrease in adipose IL-6 in obese rats compared to the untreated obese group, suggesting that treatment with melatonin mitigates inflammatory signals induced by insulin resistance/hyperinsulinemia with consequent decrease in adipose/hepatic inflammation. In consonance with previous study, melatonin acts as a free radical scavenger that eliminates reactive oxygen and promotes the action and expression of endogenous antioxidants [26, 57]. Our results are also consistent with a number of studies who have demonstrated anti-inflammatory, anti-proliferative and apoptotic properties of melatonin in experimental animals [58–60]. Nevertheless, the present results are not without limitations in such that the molecular mechanisms underlying the regulatory role of melatonin in obese animals, and the link between obestatin and other biochemical parameters were not investigated. However, the present data provide a justification for further study of molecular mechanisms, and the data perhaps, provide clinical insight into the diagnosis and management of obesity-associated adipose-hepatic metabolic comorbidities.

## 5. Conclusion

Taken together, the present results indicate that HFD exposure causes adipose-hepatic metabolic disturbance in obese animals, which are accompanied by oxidative stress and inflammation. In addition, the present results suggest that melatonin supplementation attenuates adipose-hepatic metabolic dysfunction, accompanying obesity by suppression of oxidative stress/inflammation-dependent mechanism and increasing circulating obestatin.

## Supporting information

S1 Data (XLSX)
